# The Show Must Go On: Evidence-Based Neuroanaesthesia Practices in a Tertiary Care Hospital in India During the COVID-19 Pandemic

**DOI:** 10.5152/TJAR.2021.1157

**Published:** 2022-04-01

**Authors:** Jhanvi Bajaj

**Affiliations:** Department of Anaesthesia, Seth G. S. Medical College, KEM Hospital, Mumbai, India

Dear Editor,

The management of neurosurgical patients during the coronavirus disease 2019 pandemic requires special considerations like precautions to be taken during emergency airway access in case of deterioration in neurological status, caring for critically ill patients, protecting health care workers from exposure during the surgery, and post-operative recovery stage. Through this review, we aim to provide evidence-based recommendations and expert opinions for anaesthesiologists caring for neurosurgical practices during the coronavirus disease 2019 pandemic with a focus on preparedness and good clinical neuroanaesthesia practice.

## Clinical Manifestations of COVID-19 in Neurosurgical Patients

The most common clinical manifestations of COVID-19 have been described as fever, respiratory symptoms, myalgia, and fatigue.^1^ Neurological symptoms like dizziness, headache, loss of sense of smell and taste,^2^ encephalopathy, and altered mental status^3^ have also been found to be common in patients with COVID-19. There have been many reports about acute ischemic stroke also being one of the common presentations of patients suffering from COVID-19.^2,4^ These patients may often come for endovascular treatment or open surgeries.

## Considerations for Testing Pre-operatively

The goals of COVID-19 testing specific neurosurgical patients are (a) to protect health care workers by ensuring the use of appropriate personal protective equipment (PPE) and (b) to prevent other post-operative patients from contracting the infection in the recovery room. As our hospital is surrounded by many containment zones that form the major source of our patients, we perform a preliminary temperature check and ask for a verbal history of cough or fever as part of the screening policy set by our municipal health authorities and the Indian Council of Medical Research.^5^ Patients with positive history or temperature >38˚C are then sent for real-time quantitative fluorescence polymerase chain reaction test and COVID-19-specific IgM and IgG antibody test. If found positive, these patients are admitted to the COVID-dedicated isolation rooms in the hospital which are suitable for droplet and contact precautions as recommended.^6^

## Pre-operative Anaesthesia Evaluation

Irrespective of the COVID status of the patient, the preanesthesia evaluation of all patients is done by the neuroanesthesiologist wearing full PPE. A distance of 2 m is maintained during the interaction and the patient is made to wear a surgical mask throughout. History about specific neurological symptoms related to COVID like hypogeusia and hyposmia are elicited along with other common symptoms like headache, vomiting, loss of motor or sensory power, and vision disturbances. Complete neurological examination is done (higher mental functions, cranial nerve exam, superficial and deep reflexes, and motor exam). Cough and gag reflex are not elicited while checking for cranial nerve (CN) IX and X. Special attention is paid to the function of CN I (sense of smell). In the general examination of other systems, special attention is given to inspection of the respiratory system for respiratory rate and signs of respiratory distress if any. Auscultation of all respiratory zones is performed thoroughly. Pre-operative oxygen saturation of every patient is noted during pre-operative anaesthesia evaluation. Among the pre-operative tests, chest x-ray of every patient is reviewed, or lung CT if available.^1^

## Intra-operative Management

Minimum number of operating room (OR) personnel required are allowed inside the theater, and everyone has to strictly wear PPE for every case. The patient is wheeled inside once all preparations are completely done wearing a 3 ply surgical mask and a disposable sheet covering his body. All monitors are done as per American Society of Anesthesiology (ASA) standards of monitoring.^7^ In our hospital, general anaesthesia is the anaesthesia of choice for neurosurgical procedures. We use an aerosol protection box described by Tseng and Lai^8^ for intubation and extubation around the head of the patient after explaining it to him. Patients are pre-oxygenated using nasal cannula. Rapid sequence induction is followed for every patient to prevent bag-mask ventilation as it can cause aerosolization of the virus.^9^ The most experienced anaesthetist does the intubation using a videolaryngoscope to avoid direct exposure to airway and to maintain distance between the operator’s face and the patient’s face to reduce the risk of contamination.^10^ Once the endotracheal tube is inserted, it is clamped till the circuit is attached as per advice to reduce aerosol particle dispersion.^11^ Heat and moisture exchanger (HME) filters are used between facemask and breathing circuits. Maintenance of anaesthesia is done using total intravenous anaesthesia to reduce the risk of contamination of the vaporizers. Depth of anaesthesia is maintained to prevent any bucking and accidental disconnection of the tube. The aerosol box is used again during the time of extubation. Patient’s airway is thoroughly suctioned using a closed suction system. Lignocaine 1.5 mg kg^-1^ IV is given prior to extubation to the patient to reduce extubation response and cough. After extubation, the patients’ mouth is again covered with a surgical mask.

Following surgery, the soda-lime utilized for the surgery is discarded. Disposable single-use equipment is decontaminated and sterilized. Surfaces of all medical devices are cleaned with ammonium chloride. The OR is then cleaned with sodium hypochlorite 1000 ppm and treated with hydrogen peroxide.

## Special Surgical Considerations

### Pituitary Surgeries

Emergency pituitary surgeries such as adenomas presenting with visual symptoms are approached transcranially instead of transnasally or transorally to reduce risk of viral transmission as the virus is highly colonized in the oral and nasal cavity.^12,13^

## Craniotomy

Usage of drills increases the risk of aerosol contamination of the theater milieu. Thus, all craniotomies in our institute are currently done using Gigli saw and Hudson’s brace. During the procedure, the scrub nurse irrigates the surgical site to reduce aerosol formation. 

## Conclusion

As the COVID-19 pandemic continues to progress throughout the world, neuroanaesthesiologists should educate themselves of the various difficulties related to patient care during the pandemic and they should continue to update their knowledge, practice, and skills to provide a safe environment for the patient as well as fellow health care workers.
